# ERK inactivation enhances stemness of NSCLC cells via promoting Slug-mediated epithelial-to-mesenchymal transition

**DOI:** 10.7150/thno.73099

**Published:** 2022-10-03

**Authors:** Shurui Cai, Na Li, Xuetao Bai, Lu Liu, Ananya Banerjee, Kousalya Lavudi, Xiaoli Zhang, Jihe Zhao, Monica Venere, Wenrui Duan, Junran Zhang, Meng X. Welliver, Kai He, Qi-En Wang

**Affiliations:** 1 Department of Radiation Oncology, College of Medicine, The Ohio State University, Columbus, OH 43210, USA.; 2 Comprehensive Cancer Center, The Ohio State University, Columbus, OH 43210, USA.; 3 Department of Biomedical Informatics, College of Medicine, The Ohio State University, Columbus, OH 43210, USA.; 4 Burnett School of Biomedical Sciences, College of Medicine, University of Central Florida, Orlando, FL 32827, USA.; 5 Department of Human & Molecular Genetics, Herbert Wertheim College of Medicine, Florida International University, Miami, FL 33199, USA.; 6 Department of Medical Oncology, College of Medicine, The Ohio State University, Columbus, OH 43210, USA.

**Keywords:** ERK, ERK inhibitor, Non-small cell lung cancer, Epithelial-to-mesenchymal transition, Slug, Cancer stem cell, dedifferentiation

## Abstract

**Rationale:** The mitogen-activated protein kinase pathway (MAPK) is one of the major cancer-driving pathways found in non-small cell lung cancer (NSCLC) patients. ERK inhibitors (ERKi) have been shown to be effective in NSCLC patients with MAPK pathway mutations. However, like other MAPK inhibitors, ERKi rarely confers complete and durable responses. The mechanism of tumor relapse after ERKi treatment is yet defined.

**Methods:** To best study the mechanism of tumor relapse after ERK inhibitor treatment in NSCLC patients, we treated various NSCLC cell lines and patient-derived xenograft (PDX) with ERK inhibitors and evaluated the enrichment of cancer stem cell (CSC) population. We then performed a Next-generation sequencing (NGS) to identify potential pathways that are responsible for the CSC enrichment. Further, the involvement of specific pathways was examined using molecular and cellular methods. Finally, we investigated the therapeutic benefits of ERKi treatment combined with JAK/STAT pathway inhibitor using cellular and xenograft NSCLC models.

**Results:** We found that ERKi treatment expands the CSC population in NSCLC cells through enhanced epithelial-to-mesenchymal transition (EMT)-mediated cancer cell dedifferentiation. Mechanistically, ERK inactivation induces EMT via pSTAT3-mediated upregulation of Slug, in which, upregulation of miR-204 and downregulation of SPDEF, a transcription repressor of Slug, are involved. Finally, the JAK/STAT pathway inhibitor Ruxolitinib blocks the ERK inactivation-induced EMT and CSC expansion, as well as the tumor progression in xenograft models after ERKi treatment.

**Conclusions:** This study revealed a potential tumor relapse mechanism of NSCLC after ERK inhibition through the unintended activation of the EMT program, ascertained the pSTAT-miR-204-SPDEF-Slug axis, and provided a promising combination inhibitor approach to prevent tumor relapse in patients.

## Introduction

The Mitogen-activated protein kinase (MAPK) pathway through the RAS-RAF-MEK-ERK cascades has been shown to play a critical role in regulating various cellular processes including proliferation, differentiation and stress response in both normal and cancer cells 1,2. Aberrant MAPK signaling, mainly induced by activating mutations in RAS and RAF, and its upstream activator EGFR, is commonly found in non-small cell lung cancer (NSCLC) 3. Thus, targeting RAS-RAF-MEK-ERK signaling network has been exploited for treatment of EGFR or MAPK-driven NSCLC. Indeed, FDA has approved the BRAF inhibitor dabrafenib and the MEK inhibitor trametinib to be administrated concurrently for treatment of patients with metastatic NSCLC harboring BRAF V600E mutation, as well as the KRAS inhibitor sotorasib for treatment of NSCLC patients harboring KRAS-G12C mutation.

ERK1/2 is the most downstream effector in the RAS-RAF-MEK-ERK cascade, activating mutations in RAS, BRAF, and MEK can eventually activate ERK. In addition, many patients who acquire resistance to BRAF, MEK, ALK, CDK4/6, TRKA, and EGFR inhibitors show activation of ERK 4,5. Therefore, ERK was considered as the Achilles' heel of the MAPK pathway, and attracted significant interest as a therapeutic cancer target 6. Since developed, several ERK inhibitors (ERKi) have been shown to be active against different cancers harboring RAS, BRAF, or MEK mutations, as well as cancers with developed BRAF/MEK inhibitor resistance 7-11. Despite this, as observed with other small-molecule inhibitors 12, resistance may still develop in ERKi treated patients, and tumor might also relapse after treatment cessation.

Many studies have shown that a small population of cancer cells, referred to as “cancer stem cells (CSCs)”, is responsible for tumor treatment resistance and tumor relapse 13-16. CSCs, also called tumor-initiating cells, not only possess the properties of normal stem cells, e.g., self-renewal and differentiation, but also exhibit extremely high tumorigenicity. Their multidrug resistance feature also allows them to survive most treatment including chemo- and radio-therapy. Therefore, surviving CSCs can regenerate a new tumor through their self-renewal and differentiation properties, and contribute to tumor relapse.

Based on a model of cancer cell plasticity 17,18, non-CSCs and CSCs can convert to each other and achieve an equilibrium in a tumor. However, this equilibrium can be disrupted by specific microenvironmental signals or cancer therapies, leading to a predominant non-CSC-to-CSC conversion, and an expansion of the CSC population. It has been well known that epithelial-to-mesenchymal transition (EMT) can generate CSCs by reprogramming non-CSCs 19. EMT is a crucial cellular process that facilitates polarized epithelial cells to lose their junctions and polarity, and transit toward mesenchymal cells characterized by increased motility. During the EMT process, cells repress epithelial protein expression (e.g., E-Cadherin) to reinforce the destruction of adherent junction, and upregulate expression of mesenchymal markers (e.g., N-Cadherin, Vimentin) to promote migration 20. EMT is exploited by cancer cells to transport from the primary tumor site to secondary organs, resulting in cancer metastasis 21. This transition is often orchestrated by a group of core EMT transcription factors (EMT-TFs) of the SNAIL, ZEB, and TWIST families in response to pleiotropic signals 22,23.

In this study, we demonstrated that ERKi treatment could cause expansion of the CSC population through EMT-mediated cancer cell dedifferentiation. Mechanistically, ERK inactivation induces EMT via pSTAT3-mediated upregulation of Slug. Finally, we found that the JAK-STAT pathway inhibitor Ruxolitinib could not only reverse the ERKi-induced EMT and block expansion of the CSC pool, but also impede ERKi-induced tumor relapse, providing a proof of concept for combination inhibitor approach for treating NSCLC patients harboring aberrant MAPK signaling.

## Materials and Methods

### Cell culture and chemicals

Human NSCLC cell lines A549, HCC827, H460, H2122, H2030, and H838 were obtained from American Type Culture Collection (ATCC). PDX72 primary tumor cells were isolated from a NSCLC patient-derived xenograft (PDX) established by transplanting freshly removed NSCLC tumor tissue (KRAS G12C mutation) into NSG mice subcutaneously. All cells were authenticated by ATCC using Short Tandem Repeat (STR) DNA profiling and tested for mycoplasma contamination routinely. Cells were cultured in RPMI 1640 medium (Corning) supplemented with 10% (v/v) fetal bovine serum (FBS, R&D Systems, Cat. No. S11150), 100 μg/ml streptomycin and 100 units/ml penicillin, incubated at 37°C in a humidified atmosphere with 5% CO_2_. All experiments were conducted using cells within 3-20 passages after thawing from the original stocks. Spheroid cancer cells were obtained by culturing bulk cancer cells in serum-free PromoCell 3D Tumorsphere Medium XF (PromoCell, Cat. No. C-28070) in Ultra-Low Attachment dishes (Corning) for at least 12 days. ERK1/2 selective inhibitors BVD-523 (Ulixertinib) and SCH772984, MEK inhibitors trametinib (GSK1120212) and U0126, as well as the JAK1/2 inhibitor Ruxolitinib, were purchased from MedChemExpress (MCE).

### Plasmid, siRNA, shRNA, miRNA, gene transfection and establishment of Tet On-inducible stable cell line

SlugMyc_pcDNA3 was a gift from Paul Wade (Addgene plasmid # 31698) 24. siGENOME Human *SNAI2* siRNA SMART pool was purchased from Dharmacon (Lafayette, CO). SPDEF shRNA was purchased from Millipore Sigma (shSPDEF#1: 5'-CCTGGATGAAAGAGCGGACTT-3', shSPDEF#2: 5'-CTACCTCTCCTACTTTGACAT-3'). Negative control mirVana miRNA mimic (NC-M), hsa-miR-204 mirVana miRNA mimic (miR-204-M), Negative control mirVana miRNA inhibitor (NC-I) and hsa-miR-204 mirVana miRNA inhibitor (miR-204-I) were purchased from ThermoFisher Scientific. Cells were transfected with plasmids, siRNAs, shRNAs, or miRNAs by using Lipofectamine 2000 reagent (ThermoFisher Scientific) according to the manufacture's protocol.

To establish a cell line stably expressing Tet On-inducible Slug, we subcloned Slug cDNA from SlugMyc_pcDNA3 to pLVX-TRE3G vector (TaKaRa) to construct the pLVX-TRE3G-Slug vector. A549 cells were first transduced with the pLVX-Tet3G vector (TaKaRa), selected with G418 at 500 µg/ml. The G418 resistant cells were transduced with pLVX-TRE3G-Slug followed by selection with 1 µg/ml puromycin. The successfully stable transfection was confirmed using immunoblotting after cultured in the presence of Doxycycline.

### Flow cytometry and cell sorting

NSCLC cells were trypsinized, resuspended in PBS containing 1% BSA, and stained lively with the APC-conjugated E-cadherin antibody (Miltenyi Biotec, Cat. No. 130-099-723) or APC-conjugated N-cadherin antibody (Miltenyi Biotec, Cat. No. 130-116-171) at a concentration recommended by the manufacturer. Corresponding isotype controls were used to set negative gates. Cells were either analyzed by a BD LSR II Flow cytometer, or sorted by a BD Aria III Flow Cytometer. All data were analyzed and visualized by Flowjo.

For cellular ALDH activity analysis and ALDH activity-based cell sorting, the ALDEFLUOR assay kit (STEMCELL Technologies) was used following the manufacturer's protocol with a minor modification. In brief, cells with or without drug treatment were grown to at least 70% confluence. After trypsinized, 1×10^6^/ml cells were resuspended in ALDEFLUOR buffer. Cells were then incubated with 2.5 µl ALDEFLUOR reagent per 1×10^6^ cells for 45 minutes at 37 °C. One portion of cells from each sample was treated with 5 μl ALDH inhibitor, diethylaminobenzaldehyde (DEAB, 15 μM), to set the negative gate. After incubation, cells were washed once with ALDEFLUOR assay buffer and eventually resuspended in 500 µl of ALDEFLUOR assay buffer. Samples were next subjected to a BD LSR II Flow cytometer for analysis, or a BD Aria III Flow Cytometer for sorting.

### RNA extraction and quantitative reverse transcription PCR

Total RNA was extracted from the NSCLC cells using Trizol reagent (Life Technologies, Carlsbad, CA) according to the manufacture's protocol. The first strand of cDNA was generated by the reverse transcription system (Applied Biosystems) in a 20 μl reaction containing 1.5 μg of total RNA. A 0.5 μl aliquot of cDNA was amplified by Fast SYBR Green PCR Master Mix (Applied Biosystems) in each 20 μl reaction with an QuantStudio 3 system (Applied Biosystems). Expression levels were normalized to 18S expression. The primer sequences for PCR are listed in Table S1. For miRNA detection, TaqMan™ MicroRNA Assay Kit (Thermo Fisher Scientific, Cat. No. 4427975) was used to determine the hsa-miR-204-5p expression level. The expression level was normalized to reference gene *RNU6B*.

### Immunoblotting

Whole cell lysates were prepared using SDS lysis buffer [2% (w/v) SDS, 10% (v/v) glycerol, 62 mmol/L Tris-HCl, pH 6.8, and a complete miniprotease inhibitor mixture (Roche Applied Science)]. Protein concentrations were then determined using DC Protein Assay Reagents (Bio-Rad), equal amounts of proteins were separated on an SDS-polyacrylamide gel, and transferred to a nitrocellulose membrane. Membrane was blocked with 5% nonfat milk in TBST, and incubated with appropriate antibodies (Table S2) at 4°C overnight. After extensive washing, membrane was incubated with the goat-anti-rabbit or goat-anti-mouse antibodies conjugated with HPR (Millipore Sigma, Cat. No. 12-348, 12-349) for 1 h at room temperature. After washing, the protein bands were detected with chemiluminescence.

### Immunofluorescence

Immunofluorescence staining was conducted according to our previous description 25. Briefly, NSCLC cells treated with ERKi or transfected with siRNAs were seeded and cultured on cover slips. Cells were fixed with 4% paraformaldehyde and permeabilized with 0.1%Triton X-100. After blocking with 20% normal goat serum in PBS, coverslips were incubated with the primary antibody (Table S2) overnight at 4°C. After washing 4 times with PBST, slides were incubated with goat-anti-rabbit or goat-anti-mouse IgG conjugated with FITC for 1 h at room temperature, further washed with PBST, and mounted with Vectashield mounting medium containing DAPI (Vector Laboratories, Burlingame, CA). Cell images were visualized using the Revolve fluorescent microscope (ECHO).

### Immunohistochemistry (IHC)

PDX72 tumor tissues were fixed with 3.7% formaldehyde for 48 h and embedded with paraffin. Tissue blocks were cut into 5 μm slides. IHC was performed as previously described 26 to examine expression of E-cadherin and Vimentin using the corresponding antibodies (Table S2). Slides were imaged under Revolve microscope (ECHO).

### Sphere formation assay

The sphere forming ability was assessed using a semi-solid sphere formation assay established in our previous study 26. Briefly, designated number of cells were mixed with semisolid media (MethoCult H4100; STEMCELL Technologies) in PromoCell 3D Tumorsphere Medium XF, and seeded in six-well Ultra-Low Attachment plates (Corning). Cells were maintained in a humidified incubator at 37 °C with 5% CO_2_ for 12 days. Meanwhile, 100 cells were also seeded in each well of a regular 6-well plate, cultured in the regular RPMI 1640 medium supplemented with 10% FBS for 12 days, to determine the colony formation ability of these cells. The number of tumorspheres was counted under an inverted Nikon microscope, and the sphere formation rate was normalized to the colony formation rate.

### Wound healing assay

NSCLC cells treated with compounds or transfected with siRNAs were grown to confluent in 6-well plates and scratched with a 200-μl pipette tip. Cells were washed with PBS twice and further cultured for 24 h. Cell images were taken at 0 h and 24 h for measuring the distance of the scratch. The scratch area was quantified using ImageJ and the closure rate was calculated accordingly.

### Transwell invasion assay

A Corning Biocoat Matrigel Invasion Chamber (Corning) was used to assess the cell invasion ability of NSCLC cells after ERKi treatment. Briefly, 5 × 10^4^ cells were seeded into Matrigel coated chambers and incubated for 24 h. After incubation, non-migrated cells on the upper membrane were removed with a cotton swab and migrated cells were fixed with cold methanol and stained with crystal violet. Migrated cells were counted under the Revolve microscope, and the relative invasion ability was calculated.

### RNA Sequencing and data analysis

A549 cells were treated with either BVD at 2.5 μM or DMSO for 5 days. Total RNA was isolated using Norgen Total RNA Purification Kit (Norgen Biotek) following manufacturer's instructions. After the quality control procedure, mRNA from samples were enriched using oligo(dT) beads, then fragmented randomly in fragmentation buffer, followed by cDNA synthesis using random hexamers and reverse transcriptase. cDNA library was then constructed and subjected to sequencing by Novogene. The original raw data from Illumina HiSeq were transformed to Sequenced Reads by base calling, raw reads were filtered to remove reads containing adapters or reads of low quality, and mapped to a reference genome using TopHat2 algorithm. Gene expression levels were measured by transcript abundance, and expressed as number of Fragments Per Kilobase of transcript sequence per Millions (FPKM). The differential gene expression analysis was conducted using DESeq 27. Gene Set Enrichment Analysis (GSEA) and hallmark gene sets in Molecular Signatures Database (MSigDB) were used to determine enriched pathways.

### Animal study

Animal studies were performed under the guidance of The Institutional Animal Care and Use Committee (IACUC) of the Ohio State University. Six to eight-week-old NOD *scid* gamma mice (NSG™) were purchased from The Jackson Laboratory. To determine the frequency of tumor-initiating cells (TICf) using the limiting dilution assay, designated numbers of cells (1:1 mixed with Matrigel Matrix, Corning, Cat. No. 356231, 0.1 ml total volume) were subcutaneously injected into the axilla of NSG mice. Mice were monitored for tumor initiation for up to 4 weeks post-injection, and the tumor number per group within this period was recorded to calculate the TICf using the Extreme Limiting Dilution Analysis (ELDA) software (http://bioinf.wehi.edu.au/software/elda/indew.html) 28.

To generate a NSCLC PDX model, PDX72 tumor fragments were implanted into NSG mice (male:female=1:1) subcutaneously. After tumors reached 200 mm^3^, mice were randomized into two groups, and administrated with either BVD (50 mg/kg) or vehicle reagent (DMSO) orally once every day. Tumor volume was measured every other day. Mice were treated for 10 days and sacrificed. Tumor masses were collected, weighted, and fixed for pathological analysis. To obtain primary tumor cells from the PDX72 xenograft, tumor pieces were minced and digested with collagenase in RPMI-1640 medium at 37ºC for 2 h. The digested tissue was passed through a 70-µm cell strainer to obtain single cell suspension. Excess RBC was removed by Histopaque-1077 centrifugation. The live, nucleated cells collected were seeded in culture dishes and cultured in RPMI 1640 medium supplemented with 10% FBS, 100 μg/ml Streptomycin, and 100 units/ml Penicillin.

To determine tumor relapse, A549 cells (1 × 10^6^) and HCC827 cells (1 × 10^6^) were injected subcutaneously into nude mice (male:female=1:1) to generate xenografts. After tumors reached around 5 mm in diameter, mice were randomly divided into four groups, administrated with vehicle, BVD (50 mg/kg), Ruxolitinib (100 mg/kg), or BVD+Ruxolitinib orally once every day for 9 days. Tumor volume was measured every other day. Mice were fed continuously after treatment cessation, and tumor volume was measured every day.

### Statistical analysis

The data were presented as mean ± SD as shown in the histograms or lines of all figures. Two sample t-tests or ANOVA were used to compare the difference between two groups or multiple groups, respectively. The RT-PCR data was first normalized to the internal control and then compared with the 2^^ΔCT method. Holm's procedure was used to control for multiple comparisons when needed. Linear mixed effects models were used to analyze tumor growth trends across times. For all statistical testing, P < 0.05 was considered statistically significant. All tests were two-sided.

## Results

### ERK inactivation expands the CSC population in NSCLC

**Figure 1:**
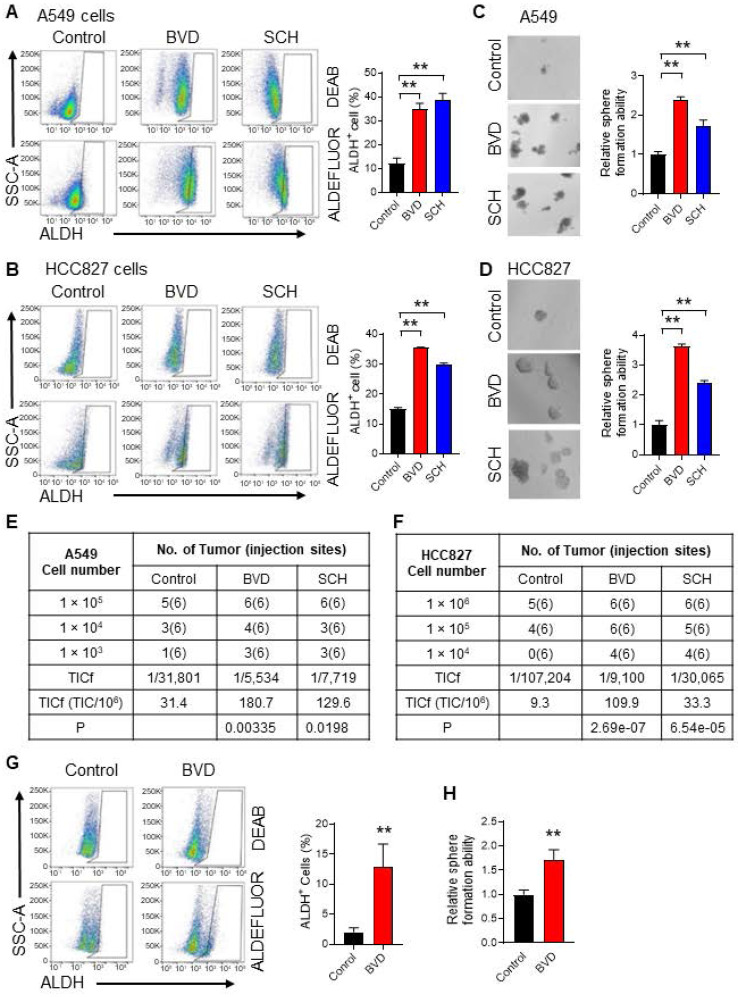
** ERK inactivation expands the CSC population in NSCLC cells.** (**A-B**) Effect of ERKi treatment on the abundance of ALDH^+^ cells in NSCLC cell lines. A549 (**A**) and HCC827 (**B**) cells were treated with BVD or SCH at 2.5 µM for 5 days. ALDH^+^ cells were assessed using the ALDEFLURE flow cytometry assay. DEAB (15 µM) treated cells were used as negative control for setting the gate. (**C-D**) Effect of ERKi treatment on the sphere-formation capacity of NSCLC cell lines. A549 (**C**) and HCC827 (**D**) cells were treated with BVD or SCH as aforementioned, the sphere-formation capacity was measured using the semisolid sphere-forming assay. (**E-F**) Effect of ERKi treatment on tumorigenicity of NSCLC cell lines. A549 (**E**) and HCC827 (**F**) cells were treated with BVD or SCH as described above, the *TICf* was quantified by a xenograft assay with limiting dilution. (**G-H**) Effects of BVD treatment on the abundance of CSCs in primary NSCLC cells. Primary tumor cells isolated from a NSCLC PDX (PDX72) were treated with BVD at 2.5 µM for 5 days; ALDH^+^ cells (**G**) and their sphere-formation capacity (**H**) were examined as described in (A-B) and (C-D). N = 3, bar: SD, **: P < 0.01.

It has been reported that suppressed ERK signaling is critical to maintenance of the self-renewal property of embryonic stem cells (ESCs) 29,30, while enhanced ERK signaling promotes the differentiation of ESCs 31. Our previous study has demonstrated that ovarian CSCs possess reduced ERK activity compared to the bulk cancer cells, and inactivated ERK signaling facilitates the maintenance of CSC properties 32. Given that ERKi are used to treat MAPK signaling-driven NSCLC, we wanted to know whether inhibition of the ERK activity can expand the CSC population. High enzymatic activity of aldehyde dehydrogenase (ALDH) is observed in CSCs and is often used to isolate and functionally characterize CSCs in NSCLC 33. We also confirmed the higher tumorigenic potential of ALDH^+^ cells compared to ALDH^-^ cells isolated from both A549 and HCC827 cell lines (Figure S1A-D). In addition, CSCs are often assessed functionally by determining their sphere forming ability *in vitro* and tumorigenicity *in vivo* 34. Thus, we determined the abundance of CSCs in NSCLC cells after ERKi treatment by analyzing these three parameters. We treated A549 and HCC827 cells, two NSCLC cell lines harboring aberrant MAPK signaling (KRAS-G12S and EGFR mutation, respectively), with two ERK inhibitors, BVD-523 (BVD) and SCH772984 (SCH), at a dose (2.5 μM) that is around their IC50 (Figure S2A-B). The inhibition of the ERK activity was also confirmed by analyzing the phosphorylated RSK (pRSK), a known ERK1/2 downstream target (Figure S2C). We found that both BVD and SCH can increase the CSC population characterized by high ALDH activity (Figure 1A-B), increase their sphere formation ability (Figure 1C-D), and enhance their tumorigenic potential (Figure 1E-F, Figure S3A-B) in these cell lines. The enrichment of ALDH^+^ cells and enhanced sphere formation ability were also found in primary tumor cells isolated from a NSCLC PDX after treated with BVD (Figure 1G-H). Furthermore, treatment of A549 and HCC827 cells with a MEK inhibitor, trametinib, increased the CSC population characterized by high ALDH activity as well (Figure S4A-D). All these data indicate that ERK inactivation indeed expands the CSC population in NSCLC.

### ERK inactivation promotes cancer cell dedifferentiation

**Figure 2:**
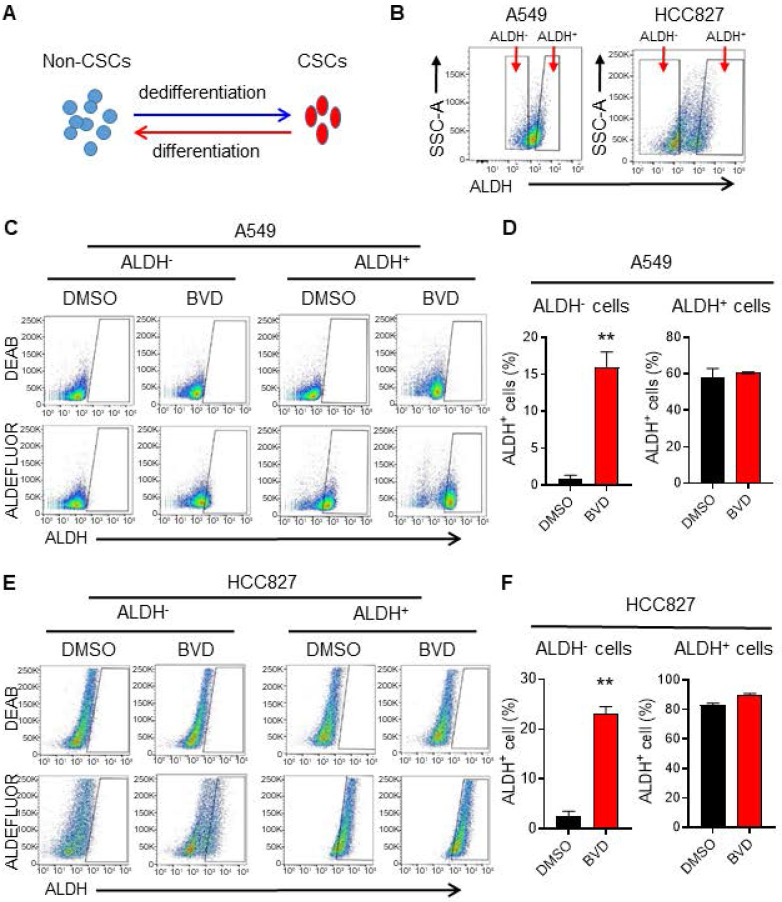
** ERK inactivation promotes dedifferentiation of NSCLC cells.** (**A**) Schematic illustration of cancer cell plasticity. (**B**) Isolation of ALDH^-^ and ALDH^+^ cells from NSCLC cell lines. A549 and HCC827 cells were incubated with ALDEFLUOR reagents, ALDH^-^ and ALDH^+^ cells were sorted using flow cytometer. (**C-F**) Conversion between ALDH^-^ and ALDH^+^ cells in the absence or presence of BVD. ALDH^-^ and ALDH^+^ cells isolated from A549 (**C, D**) and HCC827 (**E, F**) cell lines were cultured in the absence or presence of 2.5 µM BVD for 5 days. ALDEFLUOR-based flow cytometry assay was conducted to assess the percentage of ALDH^+^ cells, DEAB treated cells were used as negative control for setting the gate (**C, E**). The percentage of ALDH^+^ cells in each group was plotted (**D, F**). N = 3, bar: SD, **: P < 0.01.

Due to the cancer cell plasticity, the abundance of CSCs in tumors can be affected by CSC differentiation and non-CSC dedifferentiation 35 (Figure 2A). To determine whether inhibition of ERK activity can affect cancer cell plasticity, we sorted ALDH^-^ cells from A549 and HCC827 cells to represent non-CSCs, and ALDH^+^ cells to represent CSCs (Figure 2B) 36, and analyzed the conversion between ALDH^-^ and ALDH^+^ cells in the absence and presence of BVD. BVD treatment could significantly promote the conversion from ALDH^-^ cells to ALDH^+^ cells, while it did not influence the conversion of ALDH^+^ cells to ALDH^-^ cells in both cell lines (Figure 2C-F). Furthermore, BVD treatment can significantly enhance the expression of stemness genes (Sox2 and Oct 4 in A549 cells, Nanog in HCC827 cells), as well as the sphere formation ability in ALDH^-^ cells isolated from both A549 and HCC827 cells (Figure S5A-C). These data indicate that ERK inactivation could promote cancer cell dedifferentiation, thereby expanding the CSC population. The enrichment of the CSC population after ERKi treatment could also be a result of selective killing or growth inhibition of non-CSCs, leading to a relative increase in the CSC population. However, we found that BVD can equivalently inhibit the ERK activity and inhibit cell growth in both ALDH^-^ and ALDH^+^ cells isolated from A549 and HCC827 cells without significant induction of cell death (Figure S6A-C), excluding the possibility that the expansion of CSCs after ERKi treatment is due to selective sensitivity of non-CSCs to ERKi.

### ERK inactivation promotes EMT in NSCLC cells

**Figure 3:**
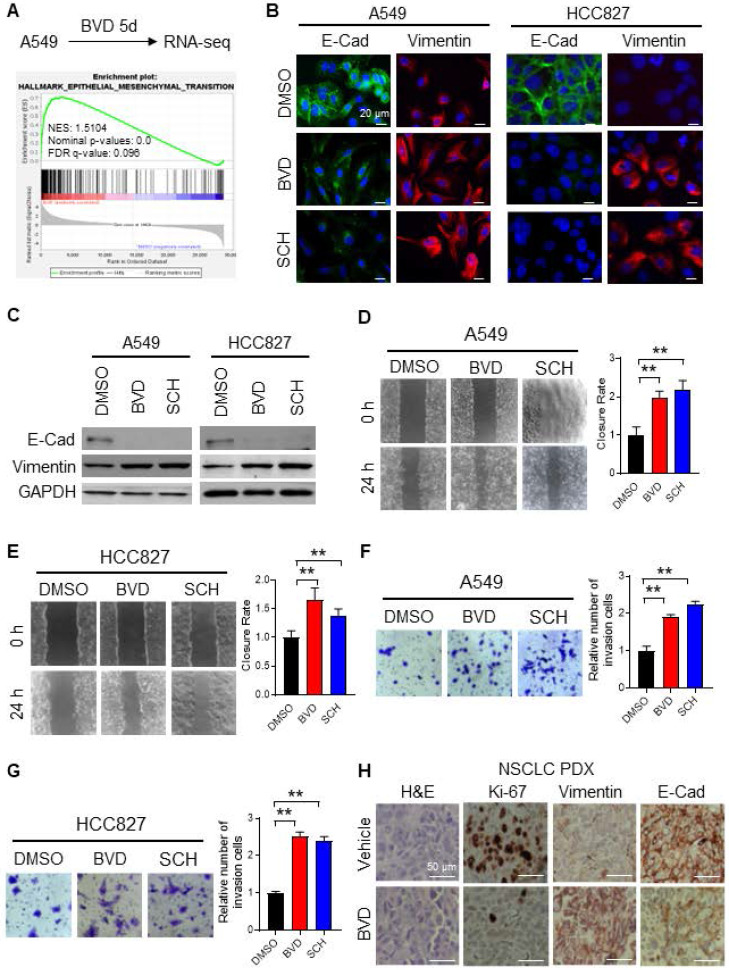
** ERK inactivation promotes EMT in NSCLC cells.** (**A**) RNA-seq and GSEA showed enrichment of EMT related genes among BVD-treated A549 cells. (**B-C**) Effect of ERKi treatment on expression of EMT markers. A549 and HCC827 cells were treated with BVD or SCH at 2.5 µM for 5 days. Immunofluorescence staining (**B**) and immunoblotting (**C**) were conducted for determining E-Cad and Vimentin expression. The pRSK level was examined to show inhibition of the ERK activity by BVD and SCH in (**C**). (**D-E**) Effects of ERKi treatment on cell migration of NSCLC cell lines. A549 (**D**) and HCC827 (**E**) cells were treated with BVD or SCH at 2.5 µM for 5 days. The migration ability of these cells was assessed using the wound healing assay. (**F-G**) Effects of ERKi treatment on cell invasion of NSCLC cells. A549 (**F**) and HCC827 (**G**) cells were treated with BVD or SCH at 2.5 µM for 5 days. The invasion ability of these cells was assessed using the transwell cell invasion assay. **H,** Effects of BVD treatment on expression of EMT markers in NSCLC tumor tissues. PDX72-bearing NSG mice were treated with BVD for 10 days, E-Cad and Vimentin expression in tumor tissues was determined using IHC. N = 3, bar: SD, **: P < 0.01.

To elucidate the mechanism behind ERK inactivation-induced cancer cell dedifferentiation and CSC enrichment, we analyzed altered gene expression caused by BVD treatment in A549 cells using RNA-seq. We have noticed a cell morphological change from epithelial morphology to elongated mesenchymal morphology when A549 cells were treated with BVD (Supplementary Figure S7A), suggesting that BVD may induce EMT. Thus, we first examined BVD-induced gene expression change of a panel of EMT biomarkers 26 in our RNA-seq data. We found that almost all mesenchymal marker genes are upregulated in A549 cells treated with BVD (Supplementary Figure S7B). Gene Set Enrichment Analysis (GSEA) further shows enrichment of the EMT hallmark gene set in BVD-treated A549 cells (Figure 3A). Next, we validated EMT in two NSCLC cell lines by analyzing expression of various EMT markers, as well as their ability of migration and invasion. Treatment with BVD and SCH was able to enhance the mRNA level of a panel of mesenchymal markers, e.g., Vimentin, N-Cadherin (N-Cad), and Fibronectin in A549 and HCC827 cells (Figure S8A-B). Furthermore, BVD and SCH-induced increase in Vimentin and decrease in E-Cadherin (E-Cad) were found in the primary tumor cells isolated from a NSCLC PDX (Figure S8C). We then confirmed the increase in the mesenchymal marker Vimentin and the decrease in the epithelial marker E-Cad in A549 and HCC827 cells after BVD and SCH treatment at the protein level (Figure 3B-C). We also validated EMT by examining the expression of E-Cad and N-Cad in BVD-treated A549 cells using flow cytometry (Figure S8D-E). The key functions of the EMT program, cell migration and cell invasion, were also enhanced by treatment with BVD or SCH in A549 and HCC827 cells (Figure 3D-G, Figure S8F-I).

To verify the ERKi-induced EMT *in vivo*, we treated mice bearing NSCLC PDXs with BVD for 10 days. As expected, BVD treatment significantly halted tumor growth (Figure S9A-B), and inhibited the proliferation of tumor cells reflected by reduced percentage of Ki-67 positive cells (Figure 3H). More importantly, the EMT program was indeed found in the surviving tumor cells, reflected by enhanced expression of Vimentin and reduced expression of E-Cad in tumors treated with BVD (Figure 3H).

Taken together, all these data indicate that ERK inactivation can promote EMT in NSCLC cells. More interestingly, we found that a MEK inhibitor, trametinib, which can eventually inhibit the ERK activity, could also enhance the mRNA level of a panel of mesenchymal markers, e.g., N-Cad and Vimentin in A549 cells, as well as N-Cad, Fibronectin, and Vimentin in HCC827 cells (Figure S4A, C, Figure S10A-B), further supporting our finding that ERK inactivation can induce the EMT program.

### Slug mediates ERK inactivation-induced EMT in NSCLC cells

**Figure 4:**
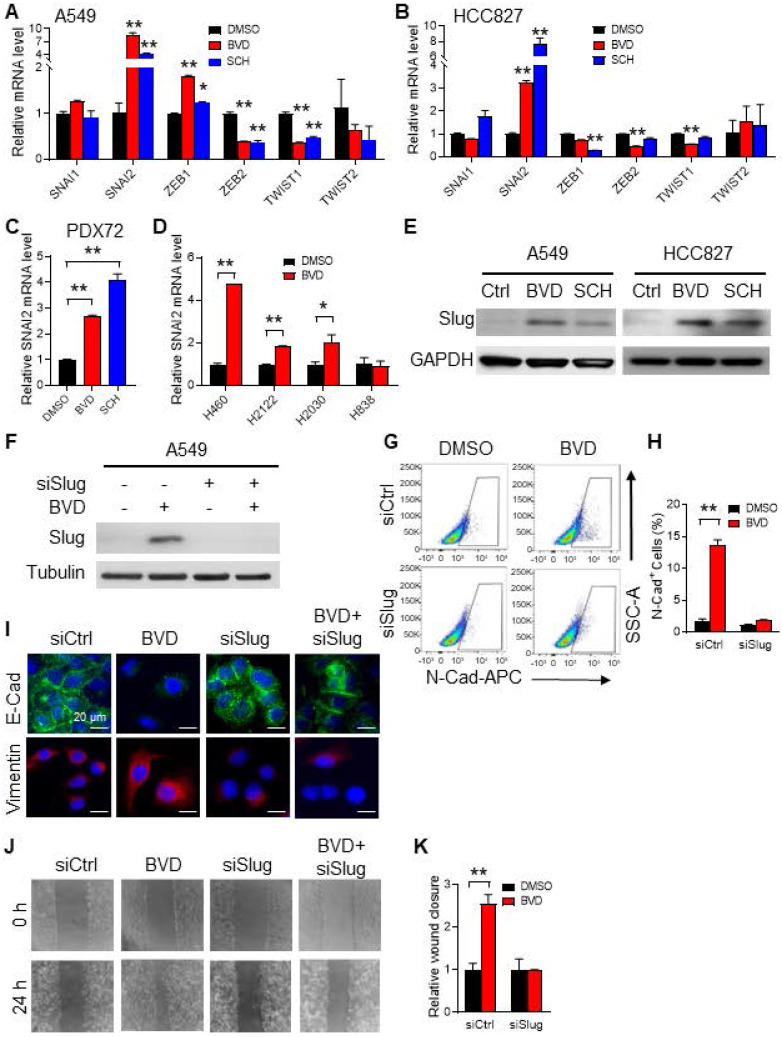
** Slug mediates ERKi-induced EMT in NSCLC cells.** (**A-B**) Effects of ERKi treatment on expression of EMT-TFs in NSCLC cell lines. A549 (**A**) and HCC827 (**B**) cells were treated with BVD or SCH at 2.5 µM for 5 days. The mRNA levels of a panel of EMT-TFs were examined using qRT-PCR. (**C**) Effect of ERKi treatment on *SNAI2* expression in primary NSCLC cells. Primary tumor cells isolated from a NSCLC PDX (PDX72) were treated with BVD or SCH at 2.5 µM for 5 days, the mRNA level of *SNAI2* was examined. (**D**) Effect of ERKi treatment on *SNAI2* expression in multiple NSCLC cell lines. H460, H2122, and H2030 cancer cells with KRAS mutation and H838 cancer cells with wild-type KRAS were treated with BVD at 2.5 µM for 5 days, the mRNA level of *SNAI2* was determined. (**E**) Effect of ERKi treatment on the protein level of Slug in NSCLC cell lines. A549 and HCC827 cells were treated with BVD or SCH at 2.5 µM for 5 days. Immunoblotting was conducted to determine the protein level of Slug. GAPDH was blotted as a loading control. (**F-I**) Effects of Slug knockdown on BVD-induced EMT. A549 cells were transfected with either control siRNA (siCtrl) or Slug siRNA (siSlug) pool for 24 h, then treated with BVD at 2.5 µM for 5 days. Immunoblotting was performed to determine the protein level of Slug (**F**). FACS was conducted to determine the presence of N-Cad^+^ cells (**G**), the percentage of N-Cad^+^ cells in each group was plotted (**H**). Immunofluorescence staining was conducted to determine E-Cad and Vimentin expression (**I**). (**J-K**) Effects of Slug knockdown on BVD-induced enhancement of the cell migration ability. siCtrl and siSlug transfected A549 cells were treated with BVD at 2.5 µM for 5 days. The wound healing assay was carried out to determine the migration ability of these cells (**J**), the relative wound closure was plotted (**K**). N = 3, bar: SD, *: P < 0.05; **: P < 0.01.

EMT is largely triggered by a set of EMT-TFs, including Snail, Slug, Twist1, Twist2, Zeb1, and Zeb2 23. To identify the EMT-TF that plays a critical role in ERK inactivation-induced EMT, we first analyzed the RNA-seq data obtained from BVD-treated and DMSO-treated A549 cells. We found *SNAI1*, *SNAI2*, *ZEB1*, and *ZEB2* were highly expressed in BVD-treated cells. Among them, *SNAI2* (encodes Slug) was the most enhanced one (Figure S7C). We further analyzed expression of these EMT-TFs in A549 and HCC827 cells after treated with BVD and SCH using qRT-PCR. Among all tested EMT-TFs, *SNAI2* was the one that is most induced by both BVD and SCH (Figure 4A-B). In addition, BVD-induced *SNAI2* expression at the mRNA level was validated in primary NSCLC cells (PDX72) and a panel of NSCLC cell lines that have the KRAS mutation, e.g., H460, H2122, and H2030, but not in H838 cells, which does not possess aberrant RAS/RAF/MEK/ERK signaling (Figure 4C-D). The induction of Slug at the protein level in A549 and HCC827 cells by both BVD and SCH treatment were confirmed using immunoblotting (Figure 4E). Interestingly, treatment with the MEK inhibitor trametinib and U0126 also induced *SNAI2* expression in A549 cells (Figure S10C-E), indicating that both direct and indirect inhibition of ERK activity can increase expression of the EMT-TF Slug (*SNAI2*).

To further characterize Slug as an EMT inducer in NSCLC cells after treatment with ERKi, we knocked down Slug expression in A549 and HCC827 cells, and analyzed expression of N-Cad after BVD treatment using flow cytometry, as well as expression of E-Cad and Vimentin using immunofluorescence. Consistently, we found that BVD increased the percentage of N-Cad^+^ cells, as well as reduced E-Cad and increased Vimentin expression. However, knockdown of Slug significantly blocked such BVD-induced EMT gene expression alteration (Figure 4F-I, Figure S11A-B). In addition, BVD-induced cell migration was also blocked by Slug knockdown in both A549 and HCC827 cells (Figure 4J-K, Figure S11C-D). Taken together, these data indicate that ERK inactivation induces the EMT program through induction of Slug expression.

### ERK inactivation upregulates Slug via the miR-204-SPDEF axis

**Figure 5:**
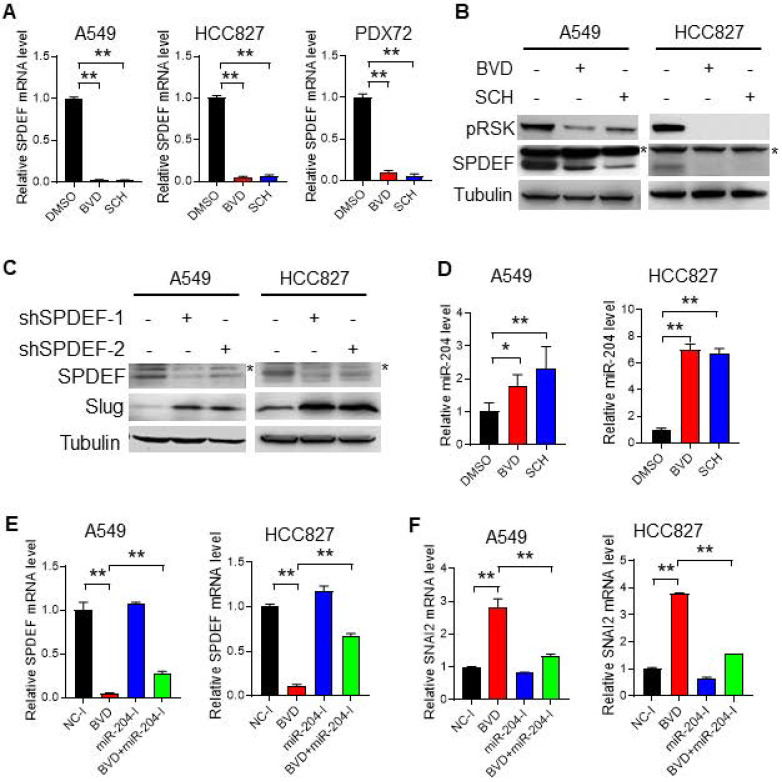
** ERK inactivation upregulates Slug *via* the miR-204-SPDEF axis.** (**A-B**) Alteration of *SPDEF* expression in NSCLC cells after ERKi treatment. A549, HCC827 cell lines and PDX72 primary tumor cells were treated with BVD or SCH at 2.5 µM for 2 days. The *SPDEF* mRNA level was examined using qRT-PCR (**A**). The SPDEF protein level in A549 and HCC827 cells were determined using immunoblotting (**B**). Arrow: non-specific band. (**C**) Effect of SPDEF knockdown on Slug expression. A549 and HCC827 cells were transfected with two different *SPDEF* shRNA for 2 days, the expression of SPDEF and Slug were determined using immunoblotting. Arrow: non-specific band. (**D**) Effect of ERKi treatment on expression of miR-204. A549 and HCC827 cells were treated with BVD or SCH at 2.5 µM for 2 days. miR-204 expression was determined using qRT-PCR. (**E-F**) Effect of miR-204 inhibition on BVD-induced downregulation of *SPDEF* and upregulation of *SNAI2*. A549 and HCC827 cells were transfected with either negative control miRNA inhibitors (NC-I) or miR-204 inhibitors (miR-204-I), treated with DMSO or BVD for 2 days. The mRNA levels of *SPDEF* (**E**) and *SNAI2* (**F**) were determined using qRT-PCR. N = 3, bar: SD, *: P < 0.05; **: P < 0.01.

Expression of Slug can be regulated by multiple mechanisms, among which, SAM pointed domain containing ETS transcription factor (SPDEF) was reported to suppress the transcription of *SNAI2* 37-39. Interestingly, our RNA-seq analysis identified SPDEF as one of the most downregulated transcription factors in A549 cells after BVD treatment (Figure S7D). Downregulation of SPDEF by ERK inactivation was further validated in NSCLC cell lines (A549 and HCC827) at both mRNA and protein levels, as well as in primary NSCLC cells (PDX72) at the mRNA level (Figure 5A-B). In addition, we further confirmed the function of SPDEF in the repression of *SNAI2* expression in A549 and HCC827 cells (Figure 5C, Figure S12). These results indicate that ERK inactivation can induce Slug expression by downregulating its transcriptional repressor SPDEF.

Next, we investigated how ERK inactivation downregulates SPDEF expression. It has been reported that SPDEF can be downregulated by miR-204 40, which was validated in A549 and HCC827 cells by transfecting them with miR-204 mimics (Figure S13). Given that miR-204 can be upregulated by BRAF inhibition 41,42, which eventually inhibits the ERK activity, we reasoned that direct ERK inhibition may also upregulate miR-204, thereby downregulating SPDEF and upregulating Slug. In support of this hypothesis, we found that both BVD and SCH could elevate the miR-204 level in A549 and HCC827 cells (Figure 5D). More importantly, miR-204 inhibitors were able to antagonize BVD-induced downregulation of *SPDEF* and upregulation of *SNAI2*, at least partially (Figure 5E, F). Taken together, these data indicate that ERK inactivation can promote miR-204-mediated depletion of SPDEF. As a transcription repressor of *SNAI2*, SPDEF downregulation derepresses the transcription of *SNAI2*, thereby enhancing Slug expression.

### Inhibition of the JAK/STAT pathway diminishes ERKi-induced EMT and prevents tumor relapse

**Figure 6:**
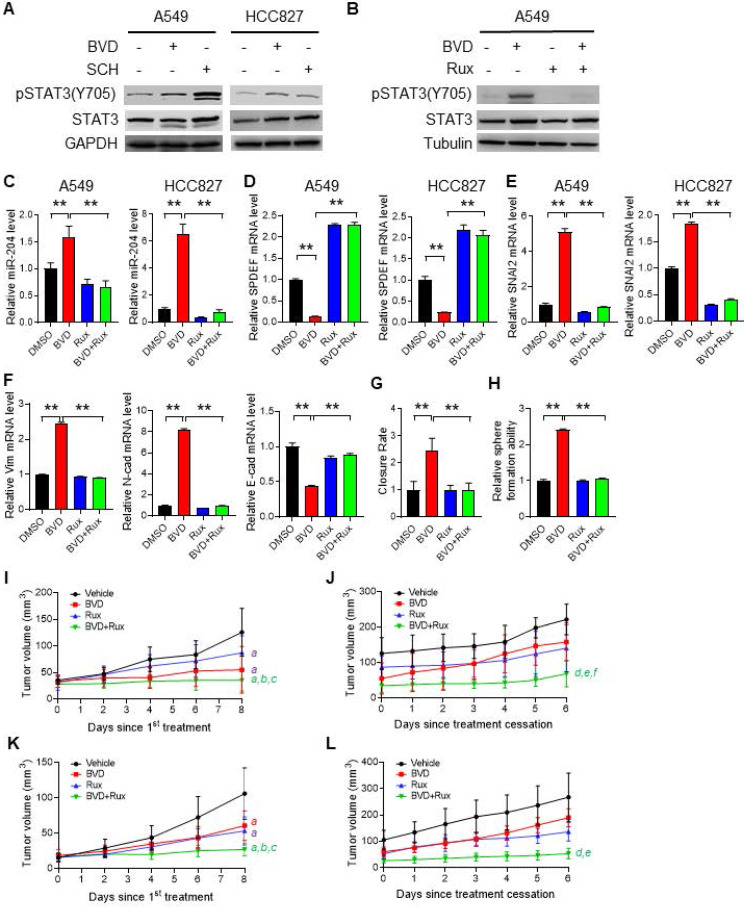
** The contribution of the JAK-STAT pathway to enhanced EMT, enrichment of CSCs, and tumor progression after ERKi treatment.** (**A-B**) Activation of JAK-STAT signaling after ERKi treatment. A549 and HCC827 cells were treated with BVD or SCH at 2.5 µM for 2 days (**A**); A549 cells were treated with 2.5 µM BVD and 10 µM Ruxolitinib (Rux) separately or in combination for 2 days (**B**). Immunoblotting was conducted to determine pSTAT3-Y705. (**C-E**) Effects of JAK-STAT inhibition on ERKi-induced alteration of miR-204, *SPDEF* and *SNAI2* expression. A549 and HCC827 cells were treated with 2.5 µM BVD and 10 µM Rux, either singly or in combination, for 2 days, mRNA levels of miR-204 (**C**), *SPDEF* (**D**) and *SNAI2* (**E**) were determined using qRT-PCR. (**F-H**) Effects of JAK-STAT inhibition on ERKi-induced EMT and CSC expansion. A549 cells were treated as in (c, d) for 5 days, the mRNA level of a panel of EMT markers were determined using qRT-PCR (**F**), the migration ability was determined using the wound healing assay (**G**), the sphere-forming capacity was determined using the semisolid sphere-formation assay (**H**). n = 3, bar: SD, **: P < 0.01. (**I-L**) Effects of JAK-STAT inhibition on tumor progression after ERKi treatment. A549 (**I, J**) and HCC827 (**K, L**) cells were injected into nude mice to generate subcutaneous xenografts. Mice were treated with BVD or Rux separately or in combination for 9 days. Tumor growth was continuously monitored for another 6 days. Tumor growth curves during treatment (**I, K**) and after treatment (**J, L**) were plotted. *a*: P < 0.001 vs. Vehicle group; *b*: P < 0.001 vs Rux group; *c*: P < 0.05 vs. BVD group; d: P < 0.001 vs Vehicle group; *e*: P < 0.001 vs BVD group; *f*: P < 0.05 vs. Rux group (Linear mixed effects models).

It has been reported that MEK, BRAF, and ERK inhibition could enhance STAT3 phosphorylation at Tyr 705 (pSTAT-Y705) 43,44, which can further increase miR-204 expression 41,42. In addition, our RNA-seq and GSEA analysis demonstrated an activated JAK-STAT3 signaling pathway in A549 cells treated with BVD (Figure S14). Given that enhanced miR-204 expression plays a critical role in ERKi-induced Slug expression, we hypothesized that inhibition of the JAK-STAT3 signaling should be able to offset the effects of ERK inactivation on EMT and CSC enrichment. In support of this hypothesis, an increased pSTAT3-Y705 was found in NSCLC cells treated with BVD and SCH (Figure 6A), whereas a blockage of BVD-induced pSTAT3-Y705 was found in cells treated with Ruxolitinib (Rux), a potent and selective JAK inhibitor (Figure 6B). We then examined whether Rux can antagonize the effect of BVD on miR-204, *SPDEF* and *SNAI2* expression, as well as on EMT and CSC expansion. Simultaneous treatment with Rux and BVD at a dose close to their IC50 (Figure S2A, Figure S15) antagonized BVD-induced upregulation of miR-204 (Figure 6C), downregulation of *SPDEF* (Figure 6D), and upregulation of *SNAI2* (Figure 6E). Furthermore, Rux also blocked BVD-induced increase in expression of the mesenchymal marker Vimentin and N-Cad, blocked BVD-induced decrease in expression of epithelial marker E-Cad (Figure 6F), as well as inhibited BVD-induced cell migration ability in A549 cells (Figure 6G), suggesting that JAK inhibition is able to offset ERKi-induced EMT. In addition, we demonstrated that Rux blocked BVD-induced augmentation of the sphere formation ability in A549 cells (Figure 6H), indicating that JAK inhibition can impede ERKi-induced enhancement of the self-renewal ability of NSCLC cells.

Finally, we generated subcutaneous xenografts in nude mice using both A549 and HCC827 cells, and determined the effect of BVD and Rux on the tumor growth during and after treatment. BVD, Rux, and BVD+Rux all significantly retard tumor growth; The BVD+Rux group showed significant reduced tumor growth rate compared to the BVD or Rux single treatment group (Figure 6I, K). When treatment was stopped, xenografts in the previously BVD-treated group started to grow at a rate like the Vehicle group. However, simultaneous treatment with BVD and Rux could significantly block tumor growth even though treatment is stopped (Figure 6J, L). All these results suggest that activation of JAK-STAT3 signaling is critical to ERK inactivation-induced EMT, enrichment of CSCs, and tumor relapse. Inhibition of the JAK-STAT3 pathway can hinder EMT program, block expansion of the CSC pool, and impede tumor progression in NSCLC cell after ERKi treatment.

## Discussion

**Figure 7:**
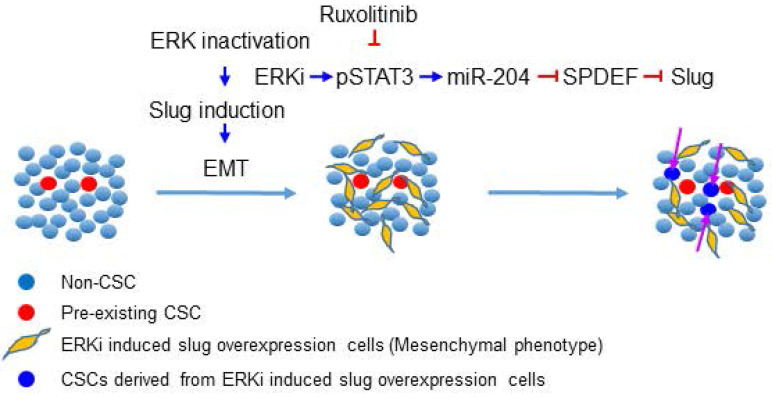
** Schematic illustration of the mechanism through which ERK inactivation expands the CSC population.** ERK inactivation activates the JAK/STAT signaling pathway, the activated pSTAT3 enhance expression of miR-204, which downregulates SPDEF expression. As a transcription repressor of Slug, SPDEF downregulation de-represses expression of Slug, triggering EMT and expanding the CSC population. The JAK inhibitor can block ERKi-induced Slug expression, thereby preventing expansion of CSCs.

ERK inhibitors have been shown to be effective in a variety of solid-tumor malignancies, but like other MAPK inhibitors, they rarely confer complete and durable responses. In this study, we revealed that ERK inactivation can expand the CSC population, which could be a contributor to tumor relapse after ERK targeted therapy. We further showed that inhibition of ERK activity triggers EMT by enhancing Slug expression via a pSTAT3-miR-204-SPDEF axis. Simultaneous inhibition of the JAK/STAT signaling could block ERK inactivation-induced EMT, expansion of the CSC population, and tumor relapse after ERKi treatment (Figure 7).

It has long been argued regarding the relationship between ERK signaling and EMT. The ERK pathway was indicated to be a mediator of EMT, mainly in non-malignant cells, such as normal murine mammary gland epithelial cells, mouse cortical tubule epithelial cells, and MCF-10A non-malignant breast epithelial cells 45-48. Prevention of TGF-β-induced EMT by MEK inhibition was also found in lung cancer cells, such as H1666 and H358 cells 49. However, other studies showed that MAPK inhibition induces EMT. For examples, the BRAF inhibitor vemurafenib upregulates EMT gene expression in BRAF or NRAS mutated melanoma cells and promotes cell invasion and metastasis 50; the BRAF inhibitor PLX4032 enhances EMT in BRAF inhibitor-resistant thyroid cancer cell line 8505C cells 51. Our study revealed that BVD and SCH, two ERK inhibitors, were able to induce EMT in NSCLC cells harboring aberrant MAPK signaling. This phenomenon was found not only in multiple cancer cell lines, but also in a NSCLC PDX. In addition, not only ERKi, but also MEKi, were shown to induce EMT in NSCLC cells. The difference between our results showing that ERKi promote EMT and the results from others showing that MEKi inhibit EMT might be due to the different cell types or cell characteristics. It has been shown that MEK inhibition increased invasion of metastatic melanoma cell lines, but reduced invasion of non-metastatic cell lines 43; BRAF inhibitor-resistant thyroid cancer cell line 8505C, but not BRAF inhibitor-sensitive BCPAP cells presented upregulated EMT following BRAF inhibitor PLX4032 treatment 51. Even though all these cells have aberrant MAPK signaling, there might be differences in other signaling pathways, such as the activated AKT pathway in cells harboring KRAS mutation 52, which may affect their responses to ERKi in modulating EMT.

Increasing evidence has shown that both CSCs and non-CSCs exhibit plasticity, which enables these cells to transit between different phenotypes when triggered, and contributes to the maintenance of the CSC population in tumors. Our data showed that ERK inhibition expands the CSC population, at least partially, by enhancing the cancer cell dedifferentiation. We also clearly demonstrated an increase in Slug expression and a Slug-mediated EMT in NSCLC cells after ERKi treatment. Cancer cell dedifferentiation, a process through which the non-CSCs acquire stemness properties, has been linked to EMT 19. In addition, overexpression of EMT-TFs, e.g., *Twist1*, *Zeb1*, or *Snai1*, can also confer the stem cell traits to normal and transformed epithelial cells as well as cancer cells 19,53-55. Thus, Slug may play a critical role in ERKi-induced cancer cell dedifferentiation via Slug-mediated EMT. However, it is also possible that Slug promotes cell dedifferentiation in an EMT-independent manner. Indeed, Slug is able to collaborate with Sox9 to induce differentiated luminal mammary epithelial cells to enter the mammary stem cells without inducing EMT, and increase the tumorigenicity of nonmetastatic breast cancer cells 53. Nevertheless, inhibition of ERKi-induced Slug expression should be able to block enrichment of CSCs after ERKi treatment.

Among all master EMT-TFs, we found ERKi specifically enhance expression of *SNAI2,* with a concurrent downregulation of SPDEF. SPDEF is a member of the ETS (E-twenty-six transformation-specific) transcription factor family. It is already known that SPDEF can block EMT by repressing the transcription of Slug in breast, hepatocellular, bladder, and prostate cancer cells 37-39. In this study, we found a dramatic decrease in SPDEF expression after ERKi treatment, and also established the negative regulation of Slug by SPDEF in NSCLC cells, further underlining the importance of SPDEF in regulating Slug and Slug-mediated EMT, and supporting the concept that SPDEF can be considered a therapeutic target 56. It has been reported that SPDEF expression can be downregulated by miR-204 40. In addition, our data demonstrated that upregulation of miR-204 is one of the contributors to the reduction of SPDEF expression in NSCLC cells after ERKi treatment. Thus, inhibition of the function of miR-204 should be able to restore the expression of SPDEF, thereby repressing Slug expression. Indeed, miR-204 inhibitors did partially antagonize BVD-induced decrease in SPDEF and increase in Slug. Therefore, halting miR-204 elevation after ERKi treatment is a key to prevent EMT and expansion of the CSC population in the treatment of NSCLC with ERKi. In this study, we also showed that downregulation of pSTAT3-Y705 with the JAK/STAT inhibitor blocked ERKi-induced EMT and expansion of the CSC population. Given that the expression of miR-204 can be upregulated by pSTAT3-Y705 in melanoma cells 41,42, these data indicate that a pSTAT3-miR-204-SPDEF axis plays an important role in regulating Slug expression after ERKi treatment. In addition, it is reported that pSTAT3 can also upregulates Slug expression directly 57. Nevertheless, given that Slug is considered “undruggable” due to inherent biological properties, inhibition of STAT3 phosphorylation at Y705 can be exploited to antagonize ERKi treatment-induced Slug upregulation, thereby blocking ERK inactivation-induced EMT, CSC enrichment, and tumor relapse. Indeed, our data have shown that inhibition of pSTAT3 with the JAK-STAT inhibitor Ruxolitinib blocked BVD-induced increase in SNAI2 expression and achieved the aforementioned aims.

It is well known that three major MAPK pathways exist in mammalian cells, the ERK1/2, the p38 kinase, and the c-JUN N-terminal kinase (JNK). A connection between ERK and JNK and/or p38 has been reported, e.g., the inhibition of ERK activity can trigger p38 kinase activation and results in activation of c-JUN N-terminal kinase (JNK) 58-60. Given that both p38 and JNK play critical roles in promoting the CSC phenotype in a variety of tumor types 61,62, it is possible that ERK inhibition may regulate the CSC population via altering the activity of p38 and JNK. Nevertheless, this putative mechanism warrants a further investigation, but does not affect the conclusion drawn from the findings in this study.

In summary, our study identified an unrecognized phenotype induced by ERK inactivation. We found that inhibition of ERK activity via ERKi leads to *SNAI2* upregulation by altering the pSTAT-miR-204-SPDEF pathway. Inhibition of the JAK-STAT pathway via Ruxolitinib abrogates ERKi-induced EMT and CSC enrichment in NSCLC cells, as well as impedes tumor progression in a xenograft model after ERKi treatment cessation. Given that Ruxolitinib has been approved by FDA for the treatment of intermediate or high-risk myelofibrosis, and its therapeutic potential in solid tumors are currently undergoing clinical evaluation, the combination of ERKi and Ruxolitinib could be exploited for treatment of NSCLC patients with aberrant MAPK signaling in the future.

## Supplementary Material

Supplementary figures and tables.
