# Supplementary material for: ERK inactivation enhances stemness of NSCLC cells via promoting Slug-mediated epithelial-to-mesenchymal transition
Source: Theranostics. 2022 Oct 3;12(16):7051–66. doi: 10.7150/thno.73099 (PMC9576621; doi:10.7150/thno.73099)
Supplement: Supplementary file 1 — Supplementary figures and tables. [file thnov12p7051s1.pdf]

ERK inactivation expands cancer stem cell population in NSCLC via promoting Slug-mediated epithelial-to-mesenchymal transition

Shurui Cai, et al

Supplementary Table

Table S1: Sequence of primers used in qRT-PCR

Primers	Sequence Forward (5'-3')	Sequence Reverse (5'-3')
SNAI2	AGATGCATATTCGGACCCAC	CCTCATGTTTGTGCAGGAGA
SNAI1	CCTCAAGATGCACATCCGAAG	ACATGGCCTTGTAGCAGCCA
ZEB1	GGCAGAGAATGAGGGAGAAG	CTTCAGACACTTGCTCACTACTC
ZEB2	CCTCTGTAGATGGTCCAGAAGA	AATTGCGGTCTGGATCGTGG
Twist1	CTGCCCTCGGACAAGCTGAG	CTAGTGGGACGCGGACATGG
Twist2	CGCTACAGCAAGAAATCGAGC	GCTGAGCTTGTCAGAGGGG
E-Cadherin	TGCCCAGAAAATGAAAAAGG	GTGTATGTGGCAATGCGTTC
N-Cadherin	CATCAAGCCTGTGGGAATCC	AATGAAGTCCCCAATGTCTCCAG
Nanog	GTCCCAAAGGCAAACAACCC	TTGACCGGGACCTTGTCTTC
SPDEF	TCTGGAAGTCAGCCTCGACC	GGGCTTGAGTAGCAACTCCTT
Vimentin	TTCCAAACTTTTCCTCCCTGAACC	TCAAGGTCATCGTGATGCTGAG
Fibronectin	CCAGTCCTACAACCAGTATTCTC	CTTCTCTGTCAGCCTGTACATC
OCT4	TCGCAAGCCCTCATTTCACC	CGAGAAGGCGAAATCCGAAG
Sox9	GAGCCGGATCTGAAGAGGGA	GCTTGACGTGTGGCTTGTTC

Table S2: Antibodies used in immunoblotting, immunofluorescence, and FACS.

Antibodies	Sources	Identifier	Dilution
Rabbit monoclonal Anti-Slug	Cell Signaling Technology	9585S	1:100 WB
Mouse monoclonal Anti-Slug	Santa Cruz Biotechnology	SC-166476	1:100 WB
Rabbit polyclonal Anti-pRSK	Cell Signaling Technology	9344S	1:1000 WB
Rabbit monoclonal Anti-RSK1	Cell Signaling Technology	8408S	1:1000 WB
Rabbit monoclonal Anti-ERK1/2	Cell Signaling Technology	4695S	1:1000 WB
Rabbit monoclonal Anti-pERK1/2 (Thr202/Tyr204)	Cell Signaling Technology	4370S	1:1000 WB
Rabbit monoclonal Anti-E-Cadherin	Cell Signaling Technology	3195S	1:1000 WB
Rabbit monoclonal Anti-Vimentin	Cell Signaling Technology	5741S	1:1000 WB<br>1:100 IF, IHC
Mouse monoclonal Anti-GAPDH	Santa Cruz Biotechnology	SC-365062	1:5000 WB
Mouse monoclonal Anti-Tubulin	Cell Signaling Technology	3873S	1:5000 WB
Rabbit polyclonal Anti-SPDEF	Proteintech	11467-1-AP	1:1000 WB
Rabbit monoclonal Anti-STAT3	Cell Signaling Technology	4904S	1:1000 WB
Rabbit monoclonal Anti-pSTAT3(Y705)	Cell Signaling Technology	9145S	1:1000 WB
Mouse monoclonal Anti-N-Cadherin-APC	Miltenyi Biotec	130-116-171	FACS
Mouse monoclonal Anti-E-Cadherin-APC	Miltenyi Biotec	130-099-723	FACS
Monoclonal REA Control-APC	Miltenyi Biotec	130-113-446	FACS
Biotinylated horse-anti-rabbit IgG (H+L)	Vector Laboratories	BA-1100	1:200 IHC
Rabbit E-Cadherin Recombinant Monoclonal Antibody	BETHYL Laboratories	#A700-088	1:100 IF, IHC
Goat anti-Rabbit IgG (H+L) Cross-Adsorbed Secondary Antibody, Alexa Fluor 594	Invitrogen	#A-11012	1:200 IF
Goat anti-Mouse IgG (H+L) Highly Cross-Adsorbed Secondary Antibody, Alexa Fluor 488	Invitrogen	#A-11029	1:200 IF
Goat Anti-Rabbit IgG Antibody, HRP-conjugate	Sigma	#12-348	1:5000 WB
Goat Anti-Mouse IgG Antibody, HRP conjugate	Sigma	#12-349	1:5000 WB

Supplementary Figures

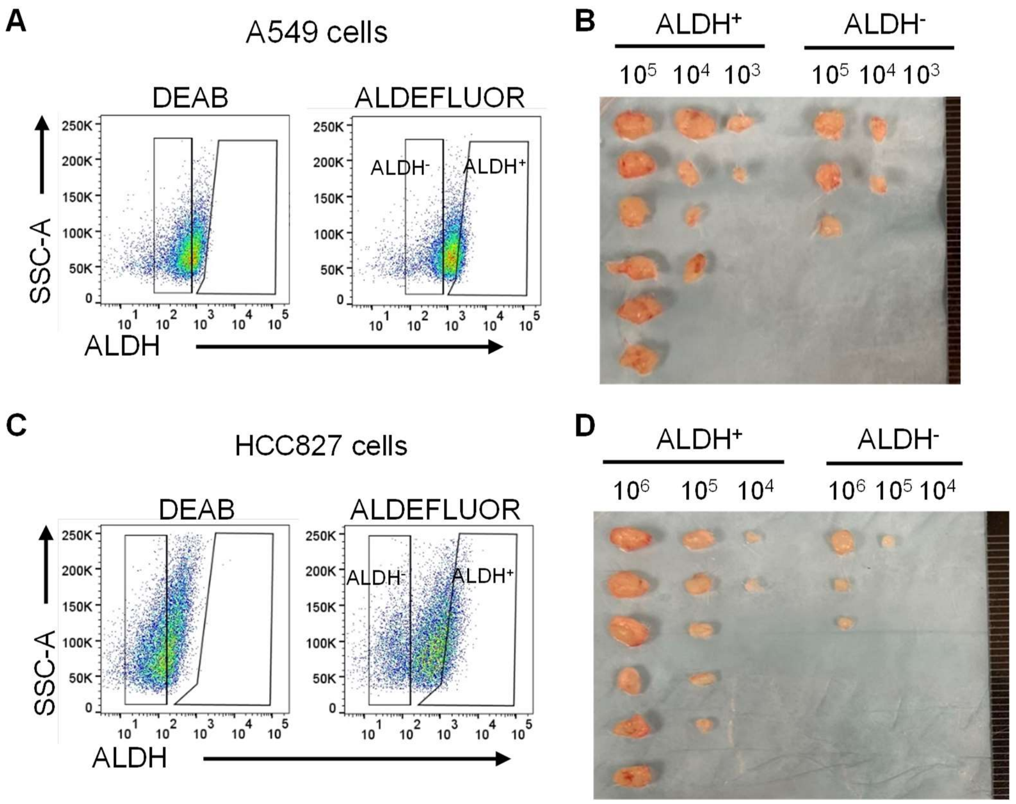

Figure S1. Determination of tumorigenicity and TICf in $ALDH^+$ and $ALDH^-$ cells sorted from NSCLC cells. $ALDH^+$ and $ALDH^-$ cells were sorted from A549 (**A**) and HCC827 (**C**) cells using the ALDEFULUOR-based FACS assay. Different numbers of cells were injected into the axilla of female NSG mice. Tumor numbers were recorded after 10 days in mice injected with A549 (**B**) and HCC827 (**D**) cells to determine the tumorigenic potential of $ALDH^+$ and $ALDH^-$ cells.

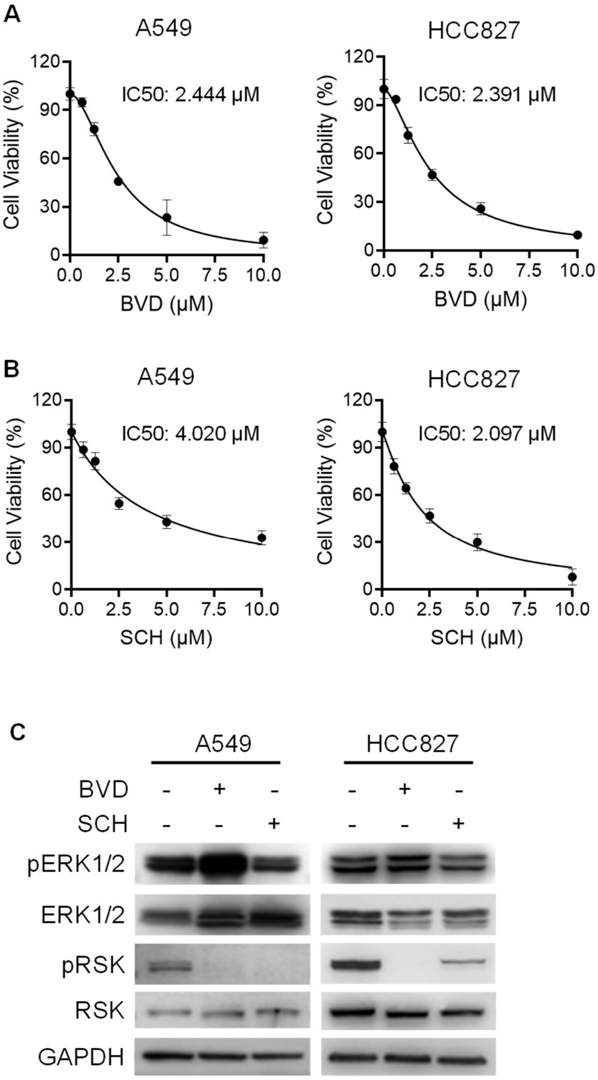

Figure S2. Evaluation of the effects of BVD and SHC on A549 and HCC827 cells. (A-B) A549 and HCC827 cells were treated with BVD (**A**) or SCH (**B**) at different doses for 7 days. The cell viability was determined using the methylene blue staining assay. The IC50 for BVD and SCH in A549 and HCC827 cells were calculated using the GraphPad. (**C)** A549 and HCC827 cells were treated with BVD or SCH at 2.5 μM for 2 days. Immunoblotting was conducted to determine pRSK to represent the inhibition of ERK. Meanwhile, pERK1/2, ERK1/2, RSK, and GAPDH were also determined to serve as control.

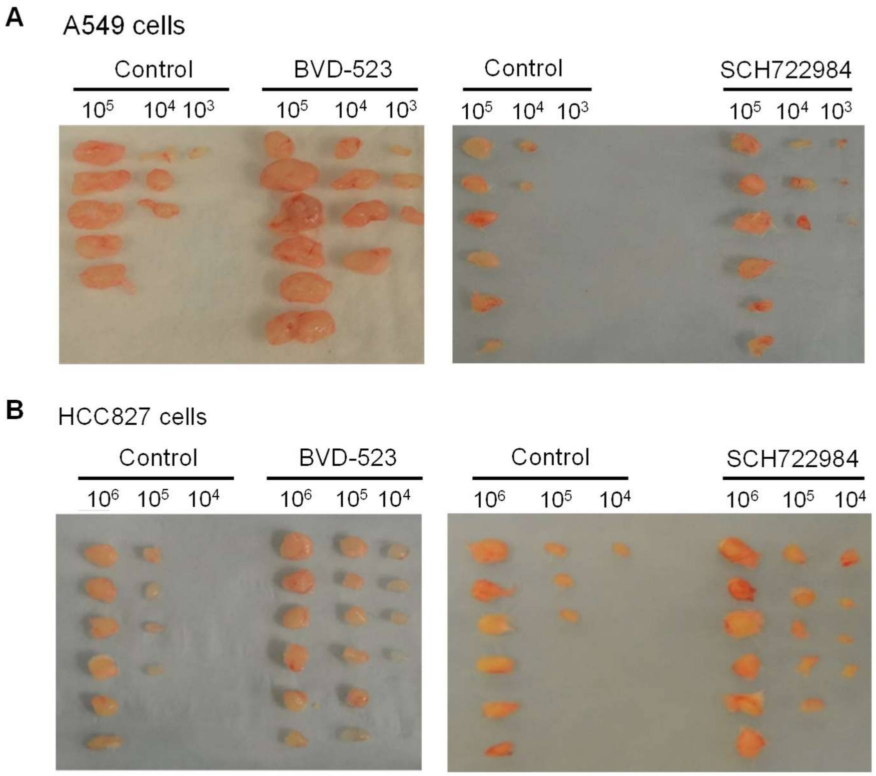

Figure S3. Determination of tumorigenicity and TICf in NSCLC cells after ERKi treatment. A549 (**A**) and HCC827 (**B**) cells were treated with BVD or SCH at 2.5 μM for 5 days. Different numbers of cells were injected into the axilla of female NSG mice. Tumor numbers were recorded after 10 days in mice injected with A549 and HCC827 cells to determine the tumorigenic potential. Images of the xenografts are shown.

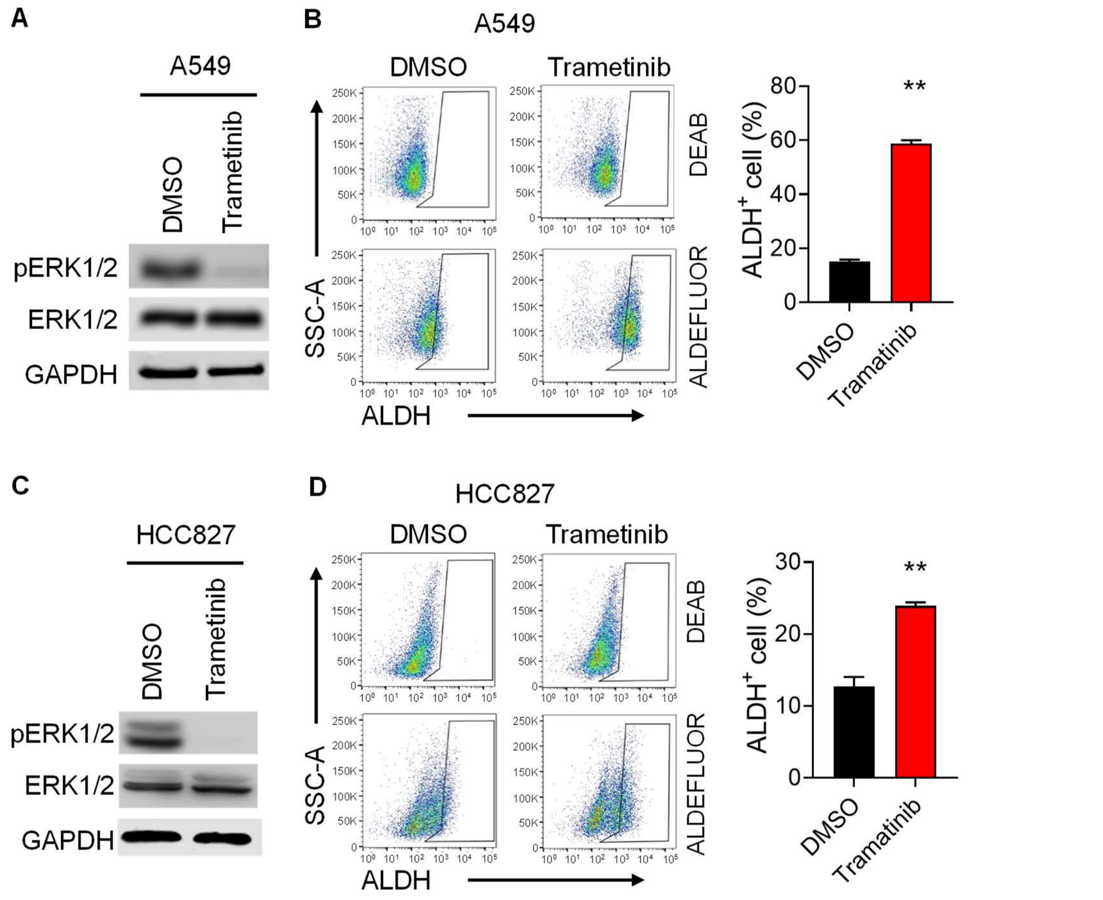

Figure S4. The MEK inhibitor enriches CSCs characterized by ALDH+ in NSCLC cells. A549 (**A-B**) and HCC827 (**C-D**) cells were treated with tramatinib at 0.5 μM for 5 days, pERK1/2 was determined using immunoblotting to show the inhibition of ERK (**A, C**); The percentage of ALDH+ cells was assessed using the ALDEFLUOR-based flow cytometry (**B, D**). DEAB treated cells were used to set negative gate. N = 3, bar: SD, **: P < 0.01.

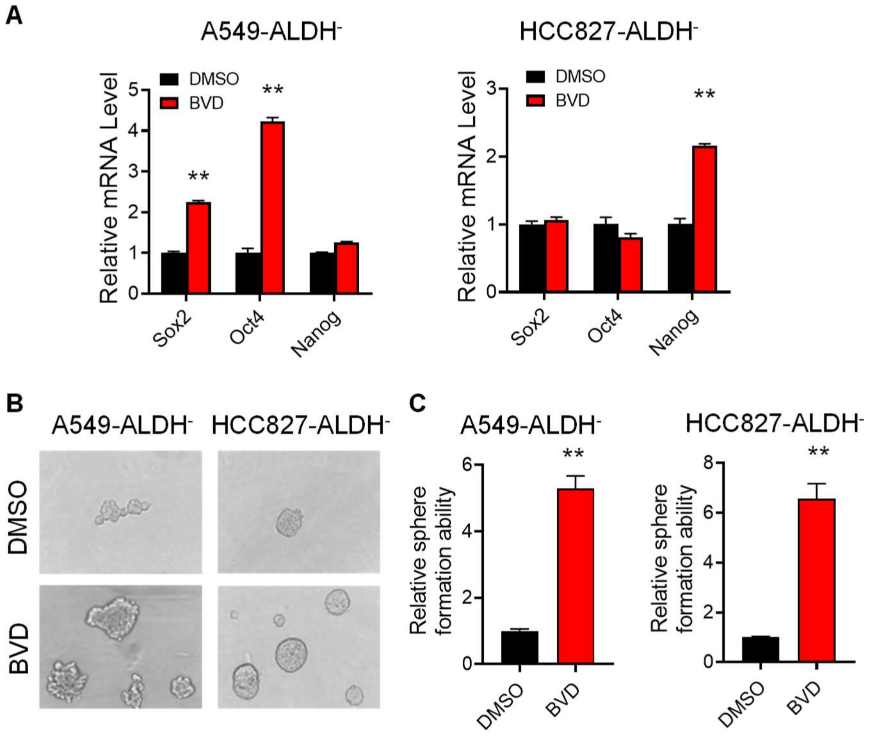

Figure S5. BVD treatment enhanced the stemness of ALDH- cells sorted from NSCLC cells. ALDH^{-} cells were sorted from A549 and HCC827 cells using the ALDEFULUOR-based FACS assay. Cells were treated with 2.5 μM BVD for 5 days. qRT-PCR was conducted to determine the expression level of stem cell-specific genes, e.g., Sox2, Oct4, and Nanog (**A**). The sphere formation assay was conducted to determine the sphere formation ability of these cells (**B-C**). N = 3, bar: SD, **: $P < 0.01$.

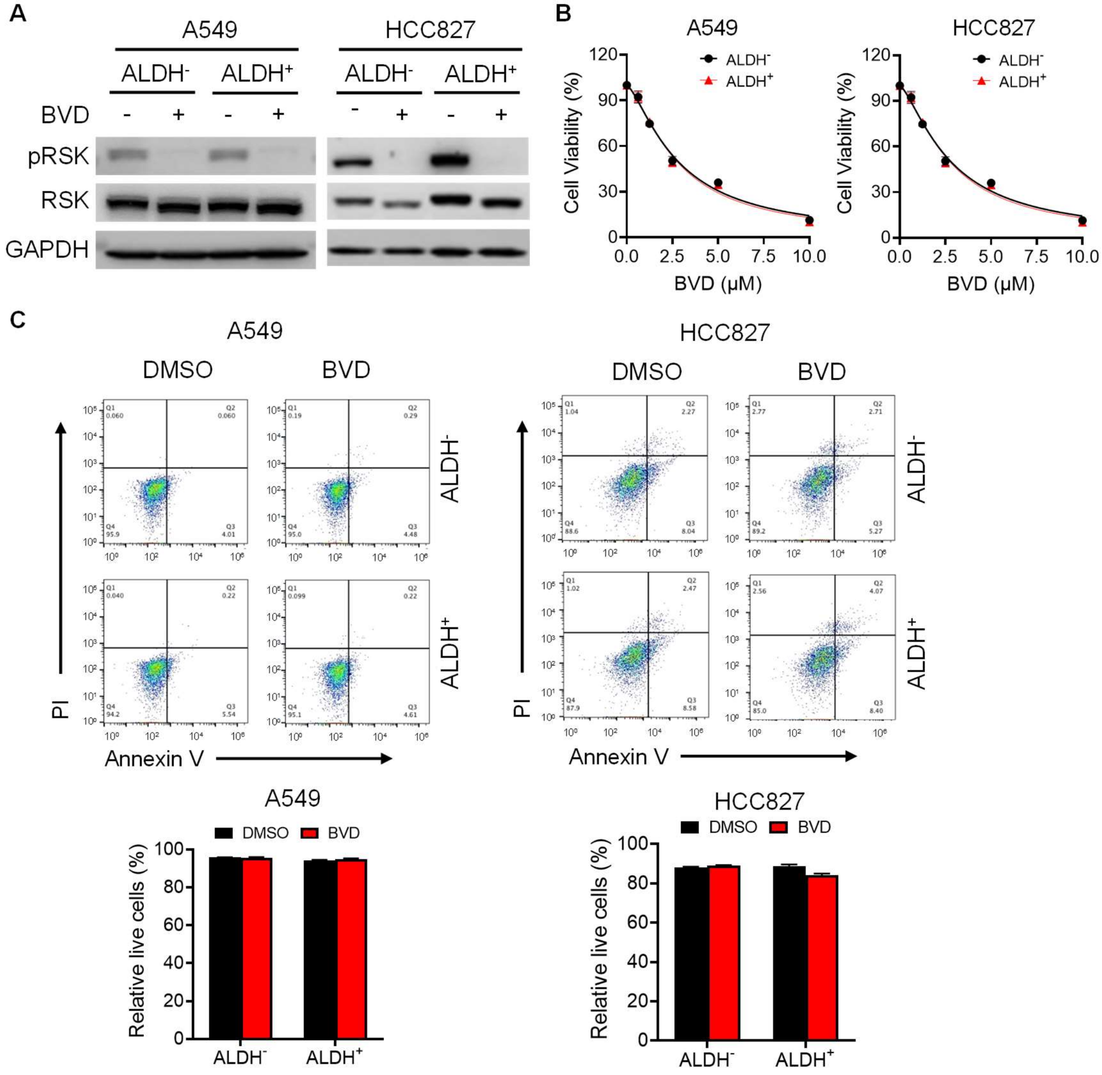

Figure S6. BVD exhibits equivalent growth inhibitory effect on ALDH$^-$ and ALDH$^+$ cells. ALDH$^-$ and ALDH$^+$ cells were sorted from A549 and HCC827 cells using the ALDEFULUOR-based FACS assay. Cells were treated with 2.5 μM BVD for 5 days. Immunoblotting was conducted to determine the expression level of pRSK to reflect the inhibition of the ERK signaling (**A**). Cells were treated with BVD at different doses for 7 days, the methylene blue staining assay was conducted to determine the cell viability (**B**). N = 5, bar: SD. Cells were treated with 2.5 μM BVD for 2 days, The Annexin V/PI staining combined with flow cytometry was conducted to determine cell death (**C**). N = 3, bar: SD.

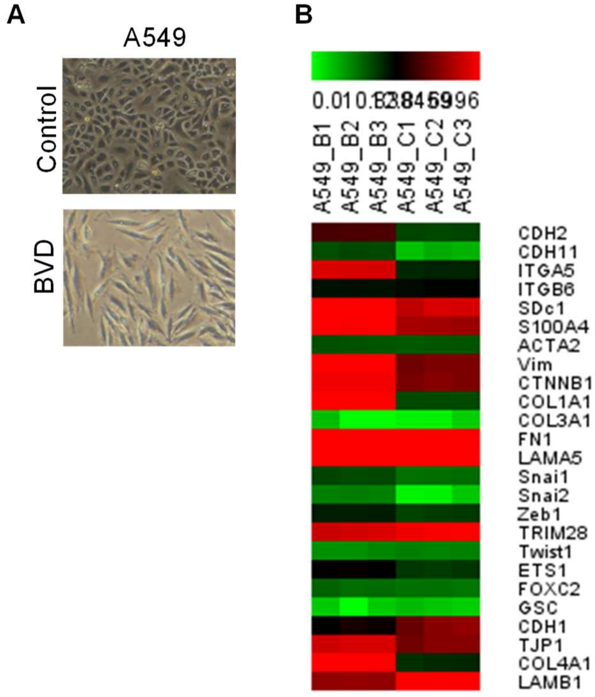

C

EMT-TF	Log2 FC (BVD vs DMSO)	P value
SNAI1	1.628932	1.29E-18
SNAI2	4.259227	2.59E-10
ZEB1	1.369674	6.10E-37
ZEB2	1.985991	9.97E-35
TWIST1	-0.53306	0.133703
TWIST2	-1.38661	1.11E-19

D

Downregulated TFs in BVD-treated cells

TF	Log2 FC (BVD vs DMSO)	P value
EGR1	-11.0603	1.27E-61
FOS	-10.7178	5.22E-56
SPDEF	-7.46042	1.54E-78
ETV4	-7.26499	6.93E-179
HMGA2	-6.63968	5.28E-27
NKX1-2	-6.46358	3.24E-07
ETV5	-6.11704	4.93E-100
HLF	-5.48587	3.92E-12
TBX4	-5.2417	5.47E-06
ETV1	-5.00834	2.01E-11

Upregulated TFs in BVD-treated cells

TF	Log2 FC (BVD vs DMSO)	P value
ZFP57	7.318974	1.08E-08
BHLHE41	6.814186	5.33E-15
MAF	6.48613	9.74E-37
ZBED2	6.277593	1.33E-12
FOXS1	5.786521	4.48E-28
MAFB	5.594134	1.09E-17
CSDC2	5.353736	4.87E-16
RUNX2	4.621314	1.43E-28
TOX	4.299712	0.030979
SNAI2	4.259227	2.59E-10

Figure S7. BVD treatment induces EMT in A549 cells. (A) Morphology change in A549 cells after BVD treatment. A549 cells were treated with either BVD at 2.5 μM or DMSO for 5 days, cell morphology was observed under microscope. (**B-D)** RNA-seq analysis shows changes in EMT-related gene expression in A549 cells after BVD treatment**.** A549 cells were treated with either BVD at 2.5 μM or DMSO for 5 days, RNA was isolated and subjected to RNA-seq analysis. Heat map of the individual gene expression changes of a panel of EMT biomarkers in BVD-treated compared to DMSO treated groups was plotted (**B**). EMT-TFs that differentially expressed in BVD-treated compared to DMSO-treated cells were listed (**C**). Top 10 downregulated TFs and top 10 upregulated TFs in BVD-treated compared to DMSO-treated cells were listed (**D**).

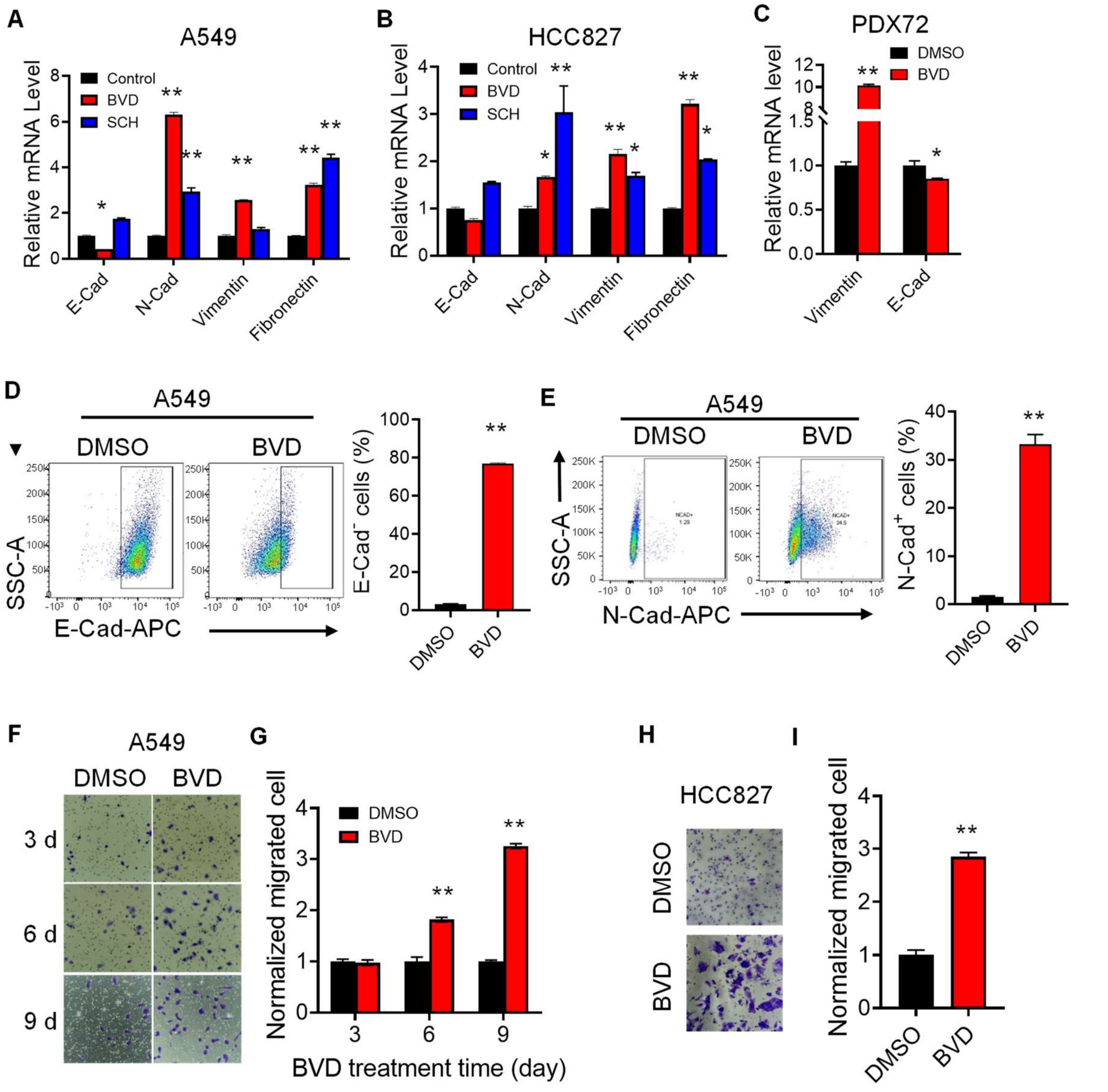

Figure S8. ERKi treatment triggers EMT in NSCLC cells. (A-B) ERKi increases the mRNA level of mesenchymal markers in NSCLC cell lines. A549 (**A**) and HCC827 (**B**) cells were treated with BVD or SCH at 2.5 μM for 5 days, qRT-PCR was conducted to examine the mRNA level of various mesenchymal markers. (**C)** BVD treatment increases expression of the mesenchymal markers primary NSCLC cells. Primary tumor cells isolated from a NSCLC PDX (PDX72) were treated with BVD at 2.5 μM for 5 days, qRT-PCR was conducted to examine the mRNA level of E-Cadherin and Vimentin. (**D-E)** BVD treatment increases the E-Cad$^-$ cell population and the N-Cad$^+$ cell population in A549 cells. A549 cells were treated with BVD at 2.5 μM for 5 days, E-Cad$^-$ and N-Cad$^+$ cells were determined using Flow cytometry. (**F-I)** BVD treatment increases the cell migration ability. A549 cells were treated with BVD at 2.5 μM for 3, 6 or 9 days (**F-G**), HCC827 cells were treated with BVD at 2.5 μM for 6 days (**H-I**), the cell migration ability was determined using the transwell cell migration assay. N = 3, bar: SD, *: $P < 0.05$; **: $P < 0.01$.

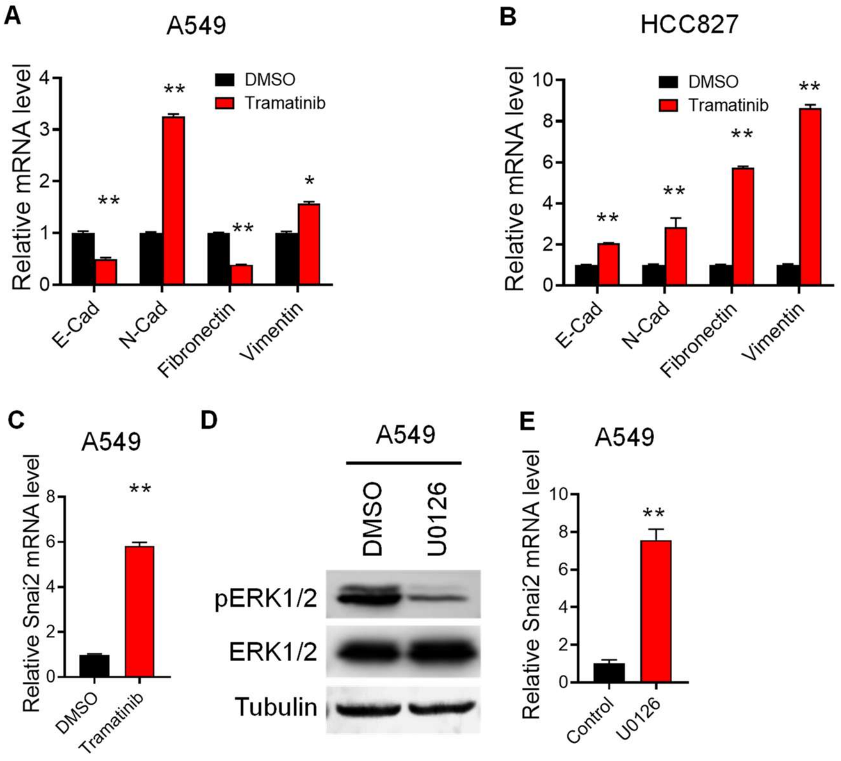

Figure S10. MEK inhibitor treatment triggers EMT and increases Slug expression in NSCLC cells. A549 (**A**) and HCC827 (**B**) cells were treated with trametinib at 0.5 μM for 5 days. Expression of EMT-related genes was examined using qRT-PCR. A549 cells were treated with trametinib (**C**) or U0126 (**D-E**) for 2 days, expression of *Snai2* was examined using qRT-PCR. Immunoblotting was conducted to confirm the inhibition of ERK1/2 by U0126 (**D**). N = 3, bar: SD, *: $P < 0.05$; **: $P < 0.01$.

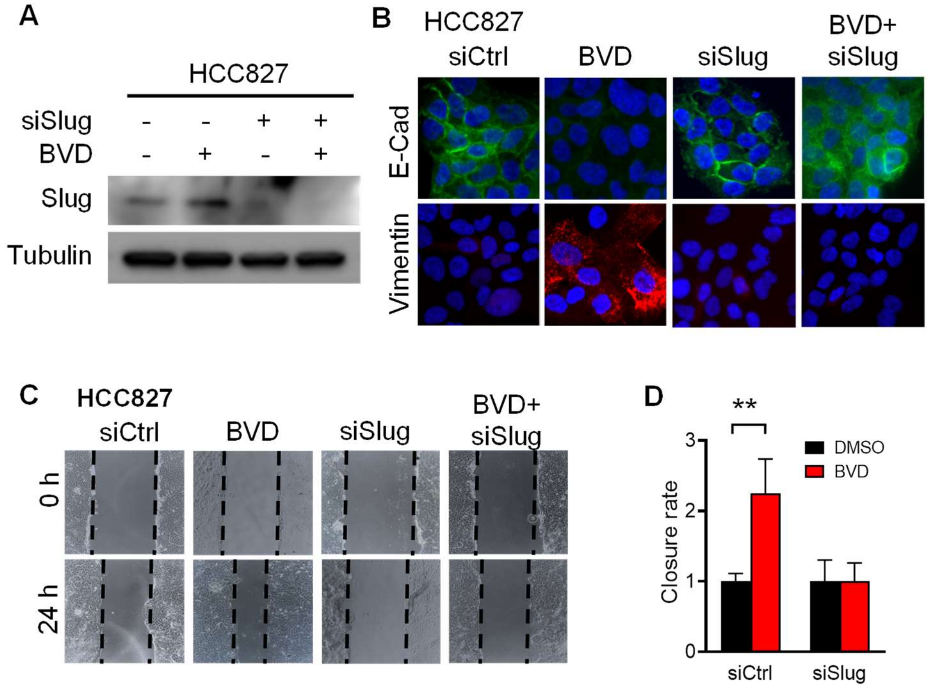

Figure S11. Slug mediates ERKi-induced EMT in NSCLC cells. HCC827 cells were transfected with either control siRNA (siCtrl) or Slug siRNA (siSlug) for 24 h, then treated with BVD at 2.5 μM for 5 days. Slug expression level was examined using immunoblotting. Tubulin was examined as a loading control (**A**). Immunofluorescence staining was conducted to determine E-Cad and Vimentin expression (**B**). The wound healing assay was carried out to determine the migration ability of these cells (**C**), the relative wound closure was plotted (**D**). N = 3, bar: SD, **: $P < 0.01$.

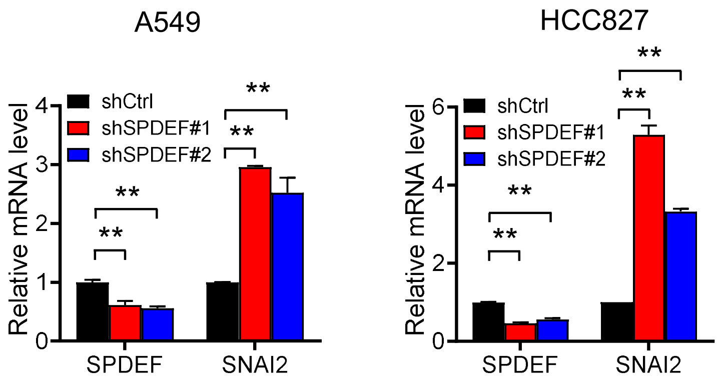

Figure S12. Knockdown of SPDEF increased the expression of SNAI2 at the mRNA level. A549 and HCC827 cells were transfected with two different shRNA targeting SPDEF for 48 h. qRT-PCR was conducted to determine the mRNA level of SPDEF and SNAI2. N = 3, bar: SD, **: $P < 0.01$.

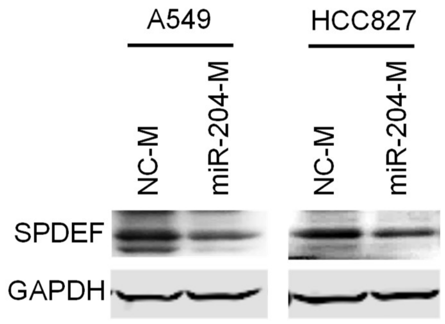

Figure S13. miR-204 downregulates SPDEF in NSCLC cells. A549 and HCC827 cells were transfected with either negative control mimic (NC-M) or miR-204 mimic (miR-204-M) for 48 h. Immunoblotting was conducted to determine the expression of SPDEF.

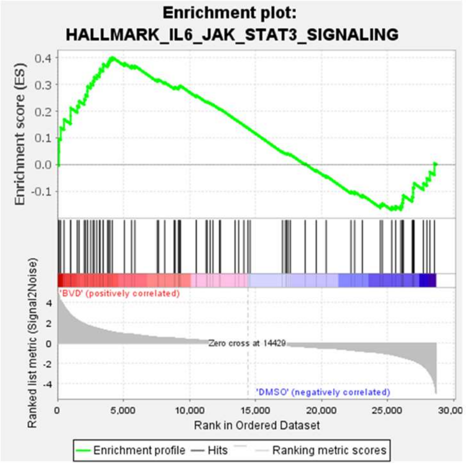

NES: 1.7279
Nominal p-value: 0.0
FDR q-value: 0.054

Figure S14. BVD treatment activates the JAK-STAT3 signaling. A549 cells were treated with either BVD at 2.5 μM or DMSO for 5 days, RNA was isolated and subjected to RNA-seq analysis. GSEA showed enrichment of JAK-STAT3 signaling related genes among BVD-treated A549 cells.

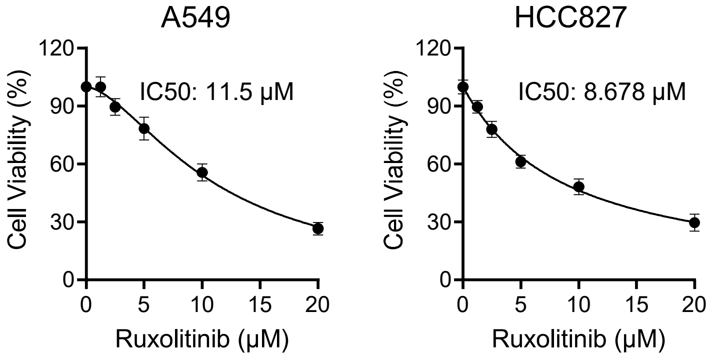

Figure S15. Determination of the IC50 of Rux in A549 and HCC827 cells. A549 and HCC827 cells were treated with Rux at different doses for 7 days. The methylene blue staining assay was conducted to determine the cell viability after treatment. The IC50 of Rux in A549 and HCC827 cells was calculated using GraphPad.
